# Inter-individual alignment of multimodal brain networks with anatomical constraints

**DOI:** 10.1162/NETN.a.514

**Published:** 2026-01-28

**Authors:** Yanis Aeschlimann, Anna Calissano, Theodore Papadopoulo, Samuel Deslauriers-Gauthier

**Affiliations:** Université Côte d’Azur, Inria, Sophia-Antipolis, France; Department of Statistical Science, University College London, London, UK

**Keywords:** Graph alignment, Inter-subject variability, Brain network, Structural connectivity, Functional connectivity, Cortical atlas

## Abstract

When using a cortical parcellation, it is generally assumed that the regions correspond across subjects, meaning regions with the same label are expected to have the same structural or functional roles from one subject to any other. However, at a fine-grained scale, environmental and genetic factors shape both cortex and white matter differently, making it challenging to consistently label all parcels for every subject. Brain networks can be constructed with regions as nodes and their connectivity as edges. One critical step while constructing those networks is the definition of the nodes due to the spatial inter-individual variability, which can limit the reliability of group studies’ results. In this work, we propose alignment criteria to register the brain nodes across subjects by allowing the permutation of tiny cortical regions. Those criteria account for multiple brain network perspectives built from different imaging modalities. Our across-subject multimodal alignment of brain networks includes constraints that restrict possible permutations to anatomically plausible ones. The identified permutations are finally applied to the brain networks not used for optimization, and also improve the alignment of these networks. This work has been validated on real magnetic resonance imaging data from the Human Connectome Project.

## Author Summary

When using a cortical parcellation, it is generally assumed that a labeled region has the same roles from one subject to any other for a structural or functional brain network’s perspective. However, at a fine-grained scale, inter-individual spatial variability due to environmental and genetic factors makes it challenging to consistently define the network nodes across subjects. A matching of the regions across subjects is proposed. It maximizes the similarity of structural and functional connectivity across subjects by permuting regions. This multimodal alignment criterion includes constraints to forbid anatomically unrealistic permutations. The identified permutations show robustness from one brain network modality to another if the proper optimization criterion with correct spatial constraints is selected.

## INTRODUCTION

Due to the large number of neurons in the brain (Herculano-Houzel, 2009), detecting whole-brain activation patterns at the neuronal scale is technically impossible with the current technologies. Therefore, an approximate but reasonable solution is to evaluate the activation of cortical regions assumed to have a homogeneous activity from the structural or functional point of view (Cabezas, Oliver, Lladó, Freixenet, & Cuadra, 2011). It is assumed that the cortical regions correspond across subjects, meaning regions with the same label are expected to have the same structural or functional roles from one subject to another. At a macroscale, the brain is organized similarly across human beings (Pessoa, 2014). Nonetheless, at a finer level, environmental and genetic factors shape differently both cortex and white matter (Thompson et al., 2001), leading to a relative spatial inter-subject variability from a structural and functional point of view (Hagmann et al., 2008; Mueller et al., 2013). As a result, it is challenging to ensure a consistent labeling of all parcels to every subject (Bohland, Bokil, Allen, & Mitra, 2009; de Reus & van den Heuvel, 2013; Dickie et al., 2017; Salehi et al., 2020). Inter-subject variability of brain activity is the topic of many studies (Mueller et al., 2013; Seghier & Price, 2009, 2018; Van Horn, Grafton, & Miller, 2008). It is notably noticed in neurosurgery, particularly for brain tumor ablations, where patients undergo highly invasive awake surgery with cortical electrical stimulation to precisely localize given functional areas (Tate, Herbet, Moritz-Gasser, Tate, & Duffau, 2014) because relying solely on atlas-predefined regions for tumor resections is not sufficient. The problem of producing a single brain atlas is exacerbated for parcellations with tiny regions.

Vigneau et al. (2006) have shown that activation peaks for language areas have a standard deviation reaching 20 mm, while the cortical atlas of 1,000 regions proposed by Schaefer et al. (2018) has a region size down to 6.0 mm. It is essential to consider this variability for studies that compare the properties of brain regions across different subjects, as we may compare mismatched regions which not represent the same structure or function, thus limiting the reliability of the results based on group analysis.

**Figure 1.**
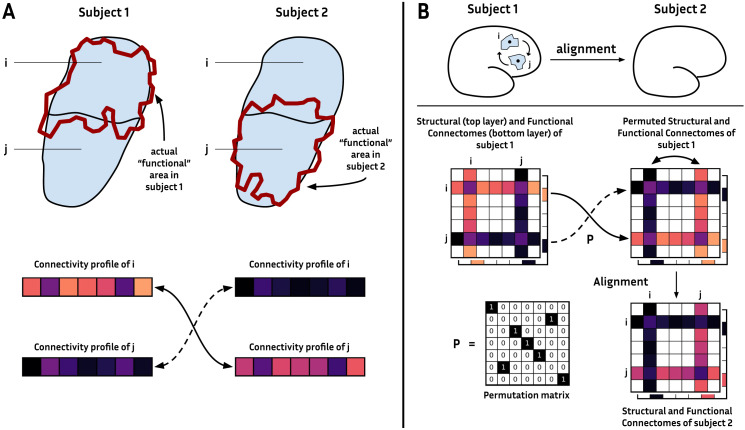
(A) Schematic example of misalignment of two predefined regions *i* and *j* on two subjects (1 and 2). Top: The ground-truth region for the same function (red outline) corresponds to Region *i* in Subject 1 and Region *j* in Subject 2. Bottom: We see a close matching of the connectivity profile between Region *i* of Subject 1 and Region *j* of Subject 2. (B) Schematic representation of the alignment of cortical regions between two subjects’ brains (top panel). The alignment of regions corresponds to permuting rows and columns of symmetric connectomes (bottom panel).

In this work, we focus on the concept of brain networks. Mathematically, a brain network is represented as a graph (Fornito, Zalesky, & Bullmore, 2016), with cortical regions and the connectivity between them being respectively the nodes and the edges of the graph (Cao et al., 2014; Sporns, Tononi, & Kötter, 2005; Yeh, Smith, Dhollander, Calamante, & Connelly, 2019). The connectivity of a region to the rest of the cortex is a unique identifier of a region within the cortex (Passingham, Stephan, & Kötter, 2002), and thus is a sufficient feature to distinguish and compare regions across different subjects (Figure 1A). The mismatching regions translate to inconsistencies in the definition of the graph nodes between subjects (Figure 1; Eickhoff, Thirion, Varoquaux, & Bzdok, 2015; Hagmann et al., 2008; Mueller et al., 2013; Sporns, 2011). Comparing brain connectivities, for instance, allows us to identify the brain regions’ homologs between species (Mars et al., 2016). A list of many methods and challenges for comparing graphs in the case of brain networks is described in Mheich, Wendling, and Hassan (2020). In a previous work, Mheich et al. (2015) measured a graph similarity across subjects and showed a significant spatial inter-subject variability in the case of EEG recordings during a picture naming task. However, they have not proposed to correct for this variability. Frigo et al. (2021) proposed an alignment algorithm to choose a suitable parcellation for many subjects. To our knowledge, only one work (Calissano, Papadopoulo, Pennec, & Deslauriers-Gauthier, 2024) has proposed to correct the inter-subject variability using the weighted structural connectivity computed from diffusion magnetic resonance imaging (MRI) by swapping the network nodes (see Figure 1A). They propose to find a matching of the regions across subjects by maximizing the graph similarity, a process well-known as graph alignment (see Conte, Foggia, Sansone, & Vento, 2004, for a review).

The work of Calissano, Papadopoulo, et al. (2024) only considers structural connectivity to align brain regions across subjects, which is a static view of the paths connecting brain regions with well-known limitations (Maier-Hein et al., 2017; Schilling et al., 2022; S. M. Smith et al., 2013; F. Zhang et al., 2022) and does not reveal the actual brain exchanges of information. A more dynamic perspective is provided by functional connectivity (van Den Heuvel & Pol, 2010) computed from resting-state functional MRI (rs-fMRI) (Biswal et al., 2010) as a correlation between the blood oxygen level–dependent (BOLD) signal of brain regions. Functional connectivity is probably coupled with structural connectivity, but it is still challenging to make the correspondence between them (Deslauriers-Gauthier, Zucchelli, Frigo, & Deriche, 2020; Smolders, De Baene, Rutten, van der Hofstad, & Florack, 2024), suggesting they bring different information about the brain networks. Furthermore, in the graph alignment proposed by Calissano, Papadopoulo, et al. (2024), the permutations of spatially close regions are initially favored in the alignment optimization, but anatomically implausible permutations of regions are not strictly forbidden.

Our work proposes a framework for a multimodal brain network alignment using simultaneously different connectivity modalities that includes a regularization term forbidding the swapping of too-distant regions to ensure anatomically plausible results. Indeed, a correct matching of regions should respect the macroscale structure of the human brain (Pessoa, 2014). The permutations identified in some brain networks are applied to brain networks from the same subjects, but not used for optimization to assess the validity of the resulting permutations. In particular, we use brain networks computed from diffusion MRI (dMRI), different fMRI runs, and paradigms. Permutations identified in one type of connectivity do not necessarily align the networks of another type of connectivity, but we show that it is possible to find permutations supported by several brain networks with our multimodal alignment and that the restriction to anatomically plausible permutations increases the robustness of the results.

## MATERIALS AND METHODS

### Connectome Alignment

To align two brain networks, we look for the best matching of nodes across the two subjects. The best matching corresponds to the one that maximizes the similarity between the permuted graph of one subject and the original graph of the subject chosen as a reference. In this work, a brain network (graph) is represented by a connectome, a *N* × *N* symmetric matrix, with *N* being the number of regions. We only consider symmetric matrices, as it is still complex to robustly evaluate the direction of the connectivity between the regions with the current MRI techniques. The values of the connectomes (i.e., the edges of the graphs) represent the connectivity strengths between the cortical regions. Swapping nodes corresponds to permuting the rows and the columns of the symmetric connectomes (see Figure 1B). Permutations can be applied on a connectome *C* by a permutation matrix *P* belonging to the permutation group 𝒫 = {*P* ∈ {0, 1}^*n*×*n*^ : *P*^*T*^**1** = *P***1** = **1**}. The permuted connectome is then *PCP*^*T*^.

The best matching of nodes across two subjects is given by a specific $\hat{P}$. To identify this $\hat{P}$, we solve the problem

$$\hat{P}=\underset{P\in 𝒫}{\text{arg}\;\text{min}}{\left\|{PCP}^{T}-C_{\text{ref}}\right\|}_{𝓕}^{2}.$$ (1)

In our case, with symmetric connectomes, the problem simplifies to

$$\hat{P}=\underset{P\in 𝒫}{\text{arg}\;\text{min}}-\text{Tr}\left({PCP}^{T}C_{\text{ref}}\right).$$ (2)

This optimization yields the permutation matrix that minimizes the distance between a corrected connectome *PCP*^*T*^ and a connectome of a subject of reference *C*_ref_. The similarity between the permuted connectome *C* and *C*_ref_ is based on the Frobenius norm ‖.‖_𝓕_. Solving this quadratic assignment problem (QAP) is NP-hard (Çela, 2013). Vogelstein et al. (2015) proposed to use the fast approximate QAP (FAQ) algorithm to tackle this difficulty by solving a relaxed version of the optimization problem in Equation 1: *P* is allowed to be in the set of bistochastic matrices 𝒟 = {*P* ∈ [0, 1]^*n*×*n*^ : *P*^*T*^**1** = *P***1** = **1**} instead of just the set of permutation matrices 𝒫. This relaxed version enables optimization by the Frank–Wolfe algorithm, also known as conditional gradient descent (Frank & Wolfe, 1956). The gradient descent should be initialized by a bistochastic matrix. At the end of the descent, the final bistochastic matrix is projected onto the set of permutation matrices (Crouse, 2016), yielding an approximate solution to Equation 1. This alignment strategy implies choosing a given connectome modality. Calissano, Papadopoulo, et al. (2024) used this algorithm on structural connectomes (SCs) to align brain networks.

### Combined Alignment of Structural and Functional Networks

To enrich the alignment of brain regions, we propose combining the information of brain networks of different modalities or types. We modified the FAQ algorithm such that it finds the permutation matrix *P* that minimizes the optimization criterion *L*_1_(*P*):

$$L_{1}\left(P\right)=\sum_{i=1}^{I}\beta_{i}{\left\|{PC}_{i}P^{T}-C_{i,\text{ref}}\right\|}_{𝓕}^{2},\;\text{subject}\;\text{to}\;\sum_{i=1}^{I}\beta_{i}=1,$$ (3)

which is, in the case of symmetric connectomes, equivalent to minimizing *L*_1_′(*P*):

$$L_{1}^{\prime }\left(P\right)=\sum_{i=1}^{I}-\beta_{i}\text{Tr}\left({PC}_{i}P^{T}C_{i,\text{ref}}\right),\;\text{subject}\;\text{to}\;\sum_{i=1}^{I}\beta_{i}=1,$$ (4)

with *C*_*i*_ and *C*_*i*,ref_ being the connectomes of a modality *i* for a subject and a subject of reference respectively. *I* is the number of connectome types taken into account. Because the different types of connectomes are built from the same atlas and considering that for a given subject, each region has the same role for all connectivity modalities, notably from a structural and functional point of view, the same permutation matrix *P* is applied to all the connectomes of the subject to be aligned. The hyperparameters *β*_*i*_ ∈ ℝ_⩾0_ allow weighting the different modalities. Indeed, if one modality is less noisy or more insightful than the others, we may favor it.

### Constraint to Specific Regions

The second contribution to the brain region alignment is the addition of a regularization term to include spatial information in *L*_1_(*P*), leading to the new criterion

$$L_{2}\left(P\right)=L_{1}\left(P\right)+\lambda \text{Tr}\left(P^{T}R\right),\;\text{subject}\;\text{to}\;\sum_{i=1}^{I}\beta_{i}=1$$ (5)

$$\;=L_{1}\left(P\right)+\lambda \sum_{k,l=1}^{N}p_{k,l}\cdot r_{k,l},$$ (6)

where *λ* is a scalar hyperparameter. *R* is a *N* × *N* regularization matrix with binary entries 0 and 1, which penalizes specific region permutations. Indeed, if *p*_*k*,*l*_ = 1 (i.e., permutation of region *l* to region *k*) and *r*_*k*,*l*_ = 1, then *L*_2_ increases by *λ*.

Dropping terms independent of *P* in *L*_2_(*P*), the problem simplifies for symmetric connectomes to minimizing *L*_2_′(*P*) defined as

$$L_{2}^{\prime }\left(P\right)=-\sum_{i=1}^{I}\beta_{i}\text{Tr}\left({PC}_{i}P^{T}C_{i,\text{ref}}\right)+\lambda \text{Tr}\left(P^{T}R\right).$$ (7)

In the FAQ algorithm, the gradient of the new *L*_2_′(*P*) optimization criterion is updated as:

$$\nabla L_{2}^{\prime }\left(P\right)=-2\sum_{i=1}^{I}\beta_{i}C_{i,\text{ref}}{PC}_{i}+\lambda R,$$ (8)

This gradient is not a bistochastic matrix, so performing the usual gradient descent by subtracting the gradient from the initialization matrix will make the resulting matrix *P* out of the bistochastic matrix space. To stay in the bistochastic matrix space, the Frank–Wolfe algorithm first computes the search direction. That is given by

$$Q_{m}=\underset{P\in 𝒟}{\arg\;\min}\langle \nabla L_{2}^{\prime }\left(P_{m}\right),P\rangle ,$$ (9)

at the iteration *m* of the gradient descent. *Q*_*m*_ is the bistochastic matrix that minimizes the scalar product with the value of the gradient of *L*_2_′(*P*_*m*_). Birkoff’s theorem (Chen, Qi, Caccetta, & Zhou, 2019) tells us that the minimum of this linear form in 𝒟 is an extremum of 𝒟, thus *Q*_*m*_ is a permutation matrix in 𝒫. With our updated gradient, the search direction *Q*_*m*_ is:

$$\begin{array}{l} Q_{m}=\underset{P\in 𝒟}{\arg\;\min}\langle \nabla L_{2}^{\prime }\left(P_{m}\right),P\rangle \\ \;=\underset{P\in 𝒟}{\arg\;\min}\text{Tr}\left(\nabla L_{2}^{\prime }{\left(P_{m}\right)}^{T}P\right)\\ \;=\underset{P\in 𝒟}{\arg\;\min}\text{Tr}\left({\left(-2\sum_{i=1}^{I}\beta_{i}C_{i,\text{ref}}P_{m}C_{i}+\lambda R\right)}^{T}P\right)\\ \;=\underset{P\in 𝒟}{\arg\;\min}\text{Tr}\left({\left(-A+\lambda R\right)}^{T}P\right),\text{with}\;A=2\sum_{i=1}^{I}\left(\beta_{i}C_{i,\text{ref}}P_{m}C_{i}\right)\\ \;=\underset{P\in 𝒟}{\arg\;\min}\sum_{k,l=1}^{N}\left(-a_{k,l}+\lambda r_{k,l}\right)\cdot p_{k,l}. \end{array}$$ (10)

The sum of Equation 10 takes values only for the *N* entries equal to 1 in *P*, so when *p*_*k*,*l*_ = 1 (*N* times). When *p*_*k*,*l*_ = 1, if *r*_*k*,*l*_ = 1, then the sum increases by *λ*, but if *r*_*k*,*l*_ = 0 the term in the sum only depends on the connectomes’ values *a*_*k*,*l*_ and not on *λ*. If the matrix *P* has its entries equal to 1 only where the matrix *R* has entries equal to 0, then no penalty (*λ* value) is added to the sum. For *λ* → ∞, if any entry *r*_*k*,*l*_ = 1 and *p*_*k*,*l*_ = 1, the regularization term takes an infinite value, and the sum cannot be minimized. It therefore forces *p*_*k*,*l*_ = 0 for any pair of indices *k*, *l*, such that *r*_*k*,*l*_ = 1. The regularization term *λ*Tr(*P*^*T*^ *R*), with *λ* → ∞, acts as a hard constraint, as *Q*_*m*_ is a permutation matrix (Chen et al., 2019) with entries equal to ones only where the entries of *R* are zeros, thus forbidding given permutations of regions. As long as it respects the constraints, the search direction *Q*_*m*_ is data-driven by the values of *a*_*k*,*l*_ in Equation 10. In other words, *Q*_*m*_ takes values that minimize the scalar product with the gradient of the optimization data criterion *L*_1_(*P*_*m*_), while still respecting the constraints imposed by *R*. Also, no arbitrary scalar value has to be chosen for *λ*. Finding the matrix *Q*_*m*_ is a linear sum assignment problem. It is computed using a version of the Hungarian algorithm by the Scipy Python package (Virtanen et al., 2020), the modified Jonker-Volgenant algorithm (Crouse, 2016). Numerically, this algorithm can handle infinite values to solve linear sum assignment problems.

The only condition in *R* for the algorithm to yield a proper permutation matrix is that there is at least a combination of entries equal to 0 in *R* that forms a permutation matrix. In our work, we furthermore use a symmetric regularization matrix *R*. If we prohibit one region from permuting to another, the latter is also forbidden from permuting to the former.

Once the search direction *Q*_*m*_ is computed, the step size *γ*_*m*_ is computed as

$$\gamma_{m}=\underset{\gamma \in \left[0,1\right]}{\arg\;\min}\;L_{2}\left(\gamma P_{m}+\left(1-\gamma \right)Q_{m}\right).$$ (11)

Since the regularization term in *L*_2_ is forced to be 0 (otherwise it takes an infinite value), it is the same to use *L*_1_ or *L*_2_ in Equation 11. The bistochastic matrix is then updated at every iteration *m* using the step size *γ*_*m*_

$$P_{m+1}=\gamma_{m}P_{m}+\left(1-\gamma_{m}\right)Q_{m}.$$ (12)

The bistochastic matrix at iteration *m* takes values between *P*_*m*_ and *Q*_*m*_, which both respect the constraints imposed by the matrix *R*. As a consequence, at every iteration, the resulting matrix is a bistochastic matrix that respects the constraints imposed by *R*. At convergence, the last bistochastic matrix is projected onto the permutation group using the modified Jonker-Volgenant algorithm (Crouse, 2016).

In this work, we chose to use SCs and functional connectomes (FCs) simultaneously, corresponding to *I* = 2. The function *L*_1_, simplifies to

$$L_{1}^{*}\left(P\right)=\alpha {\left\|{PSP}^{T}-S_{\text{ref}}\right\|}_{𝓕}^{2}+\left(1-\alpha \right){\left\|{PFP}^{T}-F_{\text{ref}}\right\|}_{𝓕}^{2},$$ (13)

while, *L*_2_ simplifies to

$$L_{2}^{*}\left(P\right)=L_{1}^{*}\left(P\right)+\lambda \text{Tr}\left(P^{T}R\right),$$ (14)

with *S* and *F* being the SCs and FCs of the subject to be aligned, and *S*_ref_ and *F*_ref_ the connectomes of the subject to align with. The values of the hyperparameter *α* can vary between 0 and 1.

### Permutation Matrix Assessment

To evaluate how a permutation matrix *P* align a pair of subject (*a*, *b*), the following error criterion is computed:

$$\eta_{i}\left(P,a,b\right)={\left\|C_{i}^{a}-C_{i}^{b}\right\|}_{𝓕}-{\left\|{PC}_{i}^{a}P^{T}-C_{i}^{b}\right\|}_{𝓕},$$ (15)

which is the difference between the connectomes’ distances $C_{i}^{a}$ and $C_{i}^{b}$ of subjects *a* and *b* for modality *i* before and after the permutations of the rows and columns using *P*. If the permutation improves the similarity between the brain networks of modality *i* of subject *a* and *b*, then *η*_*i*_(*P*, *a*, *b*) > 0. A permutation matrix resulting from the optimization of *L*_1_(*P*) or *L*_2_(*P*) does not necessarily yield *η* > 0 for both connectome types used in the optimization because the optimization is only done on a combination of the distances of the two brain network types to their respective reference connectomes. Thus, *η* is computed independently on both connectome types used in the optimization. The error criterion is also computed on connectome types not used to optimize the values of *P*, to assess the generalization of the resulting permutation matrix. We say that a permutation *generalizes* to another connectome type when optimized on a first type, it also increases the connectome similarity of connectomes built from the other type.

### MRI Acquisition and Preprocessing, HCP Data

For this study, we selected the first 600 subjects from the HCP S1200 release who underwent dMRI, rs-fMRI, and task fMRI (t-fMRI) for the seven paradigms: motor, language, social, gambling, emotion, relational, and working memory. Data were acquired using a 3T Siemens Skyra scanner (Siemens AG, Erlanger, Germany). For further information, the imaging protocols have been described in detail in Van Essen et al. (2012). The fine-grained atlas of 1,000 regions (17 networks) proposed by Schaefer et al. (2018) is used to produce the symmetric connectomes. The first 500 rows and columns of the connectomes correspond to the labels of the regions of the left hemisphere and the last 500 to the right hemisphere.

#### Functional.

The resting-state data were acquired using multiband gradient-echo echo-planar imaging (EPI). Each session consisted of two runs (with left-to-right and right-to-left phase encoding) of 14 min and 33 s each (repetition time (TR) = 720 ms, echo time (TE) = 33.1 ms, voxel size = 2 mm isotropic, number of volumes = 1,200). The task-state data were acquired using the same protocol, but the duration of acquisition per run is lower and depends on the task. Details on the protocols of t-fMRI can be found in Barch et al. (2013). Both rs-fMRI and t-fMRI are already registered in the MNI space.

Rs-fMRI data originate from the “FIX-Denoised (Extended)” dataset of HCP (S. M. Smith et al., 2013) in 4D volumetric format. This preprocessed dataset has been cleaned of structured noise through a process that pairs independent component analysis (MELODIC) with the FMRIB Software Library (FSL) tool FIX, which automatically removes artifactual or “bad” components, along with rigid-body head motion correction and high-pass temporal filtering to remove slow drifts. For further details on this method, see Griffanti et al. (2014) and Salimi-Khorshidi et al. (2014). Task-functional images were preprocessed through the HCP Minimal Preprocessing (HCP-MP) pipeline (Glasser et al., 2013), including EPI distortion correction, FLIRT-based motion correction, TOPUP-based field map preprocessing, and intensity normalization.

A Butterworth bandpass filter of order 8 with cutoff frequencies of 0.06 and 0.125 Hz is used to filter out irrelevant parts of the BOLD signal (Romascano et al., 2015). The processed BOLD signals are averaged over the regions of the atlas. A volumetric version of the Schaefer parcellation in MNI space is used. Regarding the rs-fMRI, the time series of left–right and right–left phase encoding are concatenated along the time axis, leading to time series of 29 min and 6 s. The symmetric correlation matrix is computed on processed BOLD signals using Pearson’s coefficient. This pipeline is processed on two runs of resting-state separately, leading to two resting-state FC (rs-FC) per subject, which in the following will be called rs-FC_1_ and rs-FC_2_ respectively. The same pipeline is applied to t-fMRI. A single task FC (t-FC) (Amico, Arenas, & Goñi, 2019; Cole, Ito, Cocuzza, & Sanchez-Romero, 2021; X. Zhang et al., 2013) is constructed from the seven t-fMRI time series concatenated along the time axis, leading to time series of 46 min and 56 s. This connectome represents a combination of signal BOLD correlation of the seven tasks cited previously.

#### Diffusion.

The diffusion MRI was acquired using a multiband spin-echo EPI sequence with right-to-left and left-to-right phase encoding polarities (TR = 5,520 ms, TE = 89.5 ms, voxel size = 1.25 mm isotropic). Eighteen volumes are acquired at b-value 0 s/mm^2^, 90 volumes at b-values 1,000 s/mm^2^, 90 volumes at b-values 2,000 s/mm^2^, and 90 volumes at b-values 3,000 s/mm^2^. Each volume is composed of 145 × 174 × 145 voxels.

Diffusion images were preprocessed through the HCP-MP pipeline (Glasser et al., 2013), including a zero gradient intensity normalization, EPI distortion correction using reversed-phase encoding directions, eddy-current correction, and nonlinear registration to MNI space.

We used the TractoFlow pipeline (Avants, Tustison, & Song, 2009; Di Tommaso et al., 2017; Garyfallidis et al., 2014; Jenkinson, Beckmann, Behrens, Woolrich, & Smith, 2012; Kurtzer, Sochat, & Bauer, 2017; Theaud et al., 2020; Tournier et al., 2019) to reconstruct tractograms from preprocessed dMRI. Spherical harmonics of order 10 maximum are fitted to the preprocessed dMRI data. Then, 120 seeds per voxel at the intersection of the gray matter and white matter are positioned as starting points of streamlines. The streamlines are reconstructed using a step size for particle filter tracking (PFT) of 0.3125 mm. Only valid streamlines with a length ranging from 20 to 300 mm are kept. The other parameters of the TractoFlow script are set to their default values. A structural connectome *S*′ is constructed by counting the number of streamlines included in the tractogram that connects the two regions of the given matrix entry (Yeh et al., 2019). A local search is performed to find the closest cortical region to the endpoints of the streamline, with an upper bound distance of 5.0 mm. Each SC is symmetrized by adding its transpose and dividing by 2. As the SC and FC have different units and different value ranges, we need to rescale them. To make the values of *α* meaningful for quantifying the balancing between the two modalities, we decided to rescale the SC such that it has the same Frobenius norm as the FC of the same subject, that is, *S* = $\frac{\|F{\|}_{𝓕}}{\|S^{\prime }{\|}_{𝓕}}$*S*′, with *S*′ and *F* the original symmetric SC and FC, and *S* the normalized SC. This normalization only modifies the values of SC, and thus, the FC matrices remain correlation matrices.

### Alignment Pipeline

**Figure 2.**
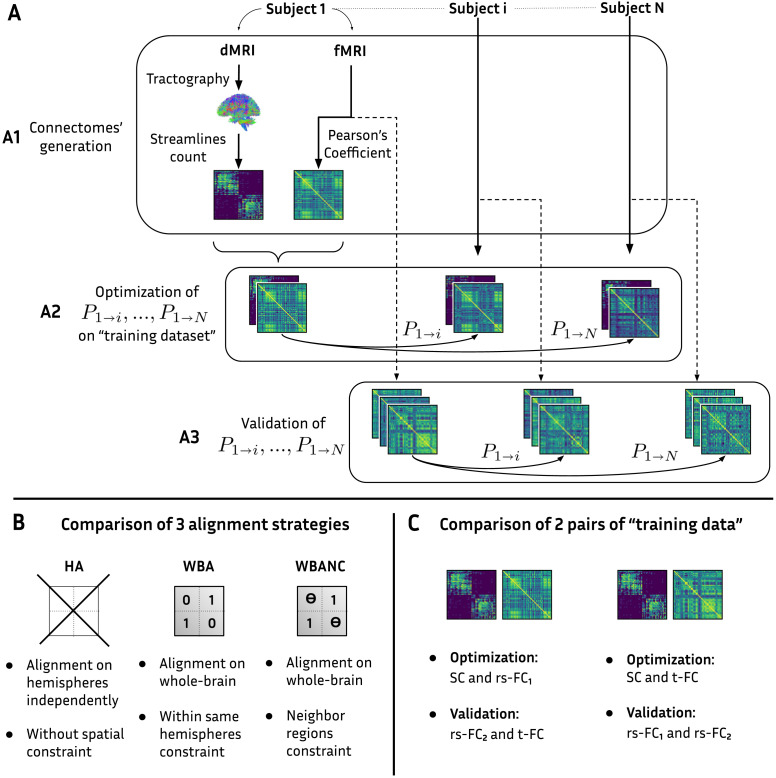
Flow chart of connectome construction from dMRI and fMRI (A1), alignment of pair of connectomes between subjects (A2), and validation of identified permutations on extra connectomes for the same group of subjects (A3). (B) Summary of the three alignment methods. (C) Summary of the connectome modalities used for the optimization (training dataset) and those used for assessment.

An overview of the alignment pipeline is illustrated in Figure 2A. The method proposed by Calissano, Papadopoulo, et al. (2024), performs the alignment on hemispheric connectomes because the proposed alignment, if performed on whole-brain connectomes, could unrealistically permute regions across the hemispheres. The alignment is carried out independently on the left and right hemispheric connectome. The whole-brain connectome is then permuted with the reconstructed *N* × *N* permutation matrix, with on its two diagonal blocks, the left and right hemispheric *N*/2 × *N*/2 permutation matrices. As a consequence, the optimization misses the across-hemisphere connectivity information. Mathematically, only the two square diagonal blocks of the whole-brain connectomes are considered (see Figure 2B).

To improve the alignment from Calissano, Papadopoulo, et al. (2024), we propose to align whole-brain connectomes while forbidding across-hemisphere permutations, so that the inter-hemispheric connectivity still informs the alignment. To forbid permutations of regions across the hemispheres, the optimization criterion $L_{2}^{*}$ of Equation 14 with the regularization term is required. Because the connectome matrices are built such that the first *N*/2 and last *N*/2 rows and columns correspond to the regions of the left and right hemispheres, respectively, the regularization matrix *R* is a 2 × 2 block matrix with equal-size blocks and values equal 0 and 1 at the diagonal and anti-diagonal blocks, respectively. We refer to this matrix as *R*_hemi_:

$$R_{\text{hemi}}=\left(\begin{array}{cc} 0 & 1\\ 1 & 0 \end{array}\right)$$ (16)

where **0** and **1** are *N*/2 × *N*/2 matrices of entries 0 and 1 respectively.

The alignment criterion with the previous constraints restricts permutations to regions within the same hemisphere. Still, it does not consider the spatial distance between the regions to perform the permutations. Regions with similar connectivity could be permuted even though they are distant, leading to anatomically implausible permutations. We propose another regularization matrix to restrict the permutations to spatially close regions. In this case, the regularization matrix refers to *R*_neigh_ defined as:

$$R_{\text{neigh}}=\left(\begin{array}{cc} \theta & 1\\ 1 & \theta \end{array}\right),$$ (17)

where ***θ*** is an *N*/2 × *N*/2 binary matrix with entries equal to 0 when the region labels corresponding to the row and column are spatially adjacent on the parcellation, and otherwise to 1. This regularization matrix aims to restrict the permutations to spatially adjacent regions. The alignment optimization using *R*_neigh_ is a subproblem of the alignment using *R*_hemi_, because the permutations allowed by *R*_neigh_ are a subset of those allowed by *R*_hemi_.

Three strategies are explored:

▪  Hemispheric Alignment (HA), using $L_{1}^{*}$ criterion from Equation 13. Two permutation matrices are independently identified for left and right hemispheric connectomes. It is the method of Calissano, Papadopoulo, et al. (2024).
▪  Whole-Brain Alignment (WBA), using $L_{2}^{*}$ criterion from Equation 14 with *R* = *R*_hemi_ defined in Equation 16.
▪  Whole-Brain Alignment With Neighborhood Constraints (WBANC), using $L_{2}^{*}$ criterion from Equation 14 with *R* = *R*_neigh_ defined in Equation 17.

Those strategies of alignment are summed up in (Figure 2B). The HA and the WBA will be compared to see the influence of the interhemispheric connectivities on the alignment results. It is interesting to check if it is still possible to find permutation matrices that are not the identity matrix when we restrict the permutations to a subset of all possible permutations, even though they are the most anatomically plausible.

The alignment requires a pair of connectomes for both the subject to be aligned and the subject to align with, leading to one permutation matrix identification. The value of *α* adjusts the identified permutation matrix as it balances the influence of the two types of connectomes in the alignment process. Therefore, for each value of *α*, one permutation matrix is identified for the alignment of two subjects (Figure 2A2). For the particular cases *α* = 0 and 1, the identified permutations do not ensure alignment of both brain network modalities, as only one modality is considered, either FC or SC. We compute the error criterion *η* seen in Equation 15 for each permutation matrix per *α* and both connectome modalities. For the specific cases, *α* = 0 and 1, this would inform us whether the identified permutations on one type of connectomes also align the other type. The permutation matrices are identified using a training dataset consisting of pairs of SC and FC. The error criterion *η* is computed on this dataset. Additionally, the permutation matrices identified for a given subject pair and a value of *α* are also applied to external connectomes from the same subjects. The error criterion is also computed on this external dataset to assess whether the alignment improves similarity on unseen data (Figure 2A3).

In this work, we use two training datasets and a different validation dataset for each of those (Figure 2C). Permutation matrices are

▪  first, identified on the pair SC and rs-FC_1_ (Training Dataset 1) and assessed on rs-FC_2_ and t-FC,
▪  secondly, identified on the pair SC and t-FC (Training Dataset 2) and assessed on rs-FC_1_ and rs-FC_2_.

In practice, as with standard registration methods where a template brain or a specific brain subject is selected as the reference to which all others are registered, one can similarly choose a random subject as the reference to align with, using the connectivity of the regions of the chosen subject as the region connectivity template. In our study, each subject is iteratively selected as a reference to all other connectomes. The gradient descent is initialized with the identity matrix, corresponding to performing no permutations. The optimization is stopped after convergence (in our case, 50 iterations). This number has been determined experimentally, as the criterion function has almost always reached a minimum after about 15 iterations. The matrices *P* are identified for *α* from 0 to 1 with a step size of 0.1 and *λ* = ∞. The 600 subjects are aligned to each other, for 11 values of *α*, the 3 strategies, and the two training datasets, leading to 23,720,400 alignment of connectomes.

A Wilcoxon signed-ranks test is used to assess the significance of the values of *η*. A statistically significant result indicates that the differences of the connectome distances before and after the permutations have stochastically greater values than a distribution symmetric about 0, which is equivalent to testing whether the distances after alignment have values stochastically lower than the ones of the distances before the alignment. We use the Bonferroni correction to compensate for the 264 hypotheses tested, corresponding to the 11 values of *α*, the three strategies, the four connectome types, and the two training datasets. A result is then considered significant if *p* < $\frac{0.001}{264}$ = 3.78 × 10^−6^.

## RESULTS

### Validation of the Algorithm on Simulated Data

To validate the alignment algorithm, we simulated 15 random permutation matrices. Those permutation matrices are applied to real connectomes of 10 randomly selected subjects. The connectomes to align are the permuted connectomes, and the respective references are the original unpermuted connectomes of the same subjects. Since the FAQ algorithm is not optimal (Vogelstein et al., 2015), we cannot guarantee the exact retrieval of the simulated permutation matrices (more precisely, we would retrieve the inverses of the simulated permutation matrices). We ran these alignments with simulated data for the HA, WBA, and WBANC algorithms and *α* = 0.5. The simulated permutation matrices were constructed to satisfy the constraints imposed by the WBA and WBANC algorithms, respectively. Using the HA strategy, in the retrieved permutations, on average, only 3.56 labels are not matched to their original labels over the 1,000 labels of the parcellation, and 58% of the resulting permutation matrices are exactly the original ones. Using the WBA and WBANC algorithms, on average, 3.32 and 1.21 labels are not matched to their original labels. Fifty-four percent and 61% of the resulting permutation matrices are exactly the original ones for WBA and WBANC. The nonoptimality characteristic of the FAQ algorithm is detailed in Vogelstein et al. (2015).

### Alignment of Real Connectomes

**Figure 3.**
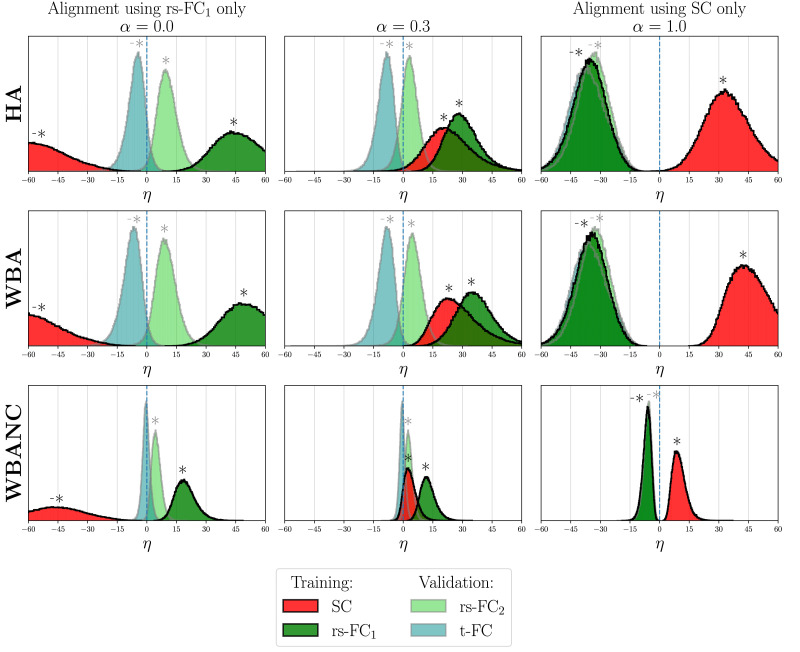
Distributions of the difference between the connectomes’ distances respectively after and before the alignment on structural and rs-FCs of the first run, for the three strategies: HA (top row), WBA (middle row), and WBANC (bottom row) and *α* = 0 (left column), 0.3 (middle column), and 1 (right column).

**Figure 4.**
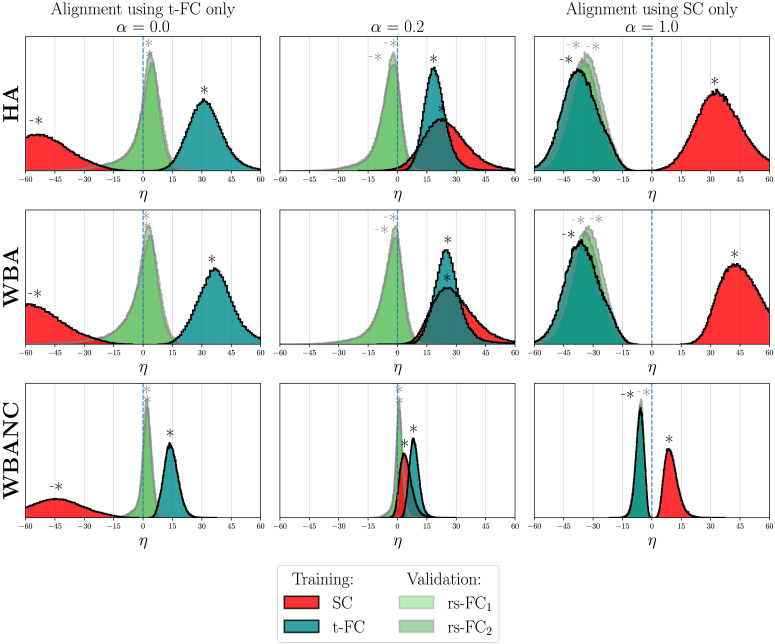
Distributions of the difference between the connectomes’ distances respectively after and before the alignment on structural and task FCs, for the three strategies: HA (top row), WBA (middle row), and WBANC (bottom row) and *α* = 0 (left column), 0.2 (middle column), and 1 (right column).

As explained in the Alignment Pipeline subsection, the error criterion *η* is computed on the connectomes of the training datasets using the *P* identified for each discretized value of *α* between 0 and 1. The criterion *η* is also computed on connectomes not used by the optimizations to assess the validity of the identified permutations. Results are shown in Figures 3 and 4 for each of the two training datasets, respectively, and the three strategies HA (first row), WBA (second row), and WBANC (third row). Only results for three values *α* are shown: *α* = 0, corresponding to an alignment on the FC only (either rs-FC_1_ or t-FC); *α* = 1, corresponding to an alignment on SC only; and the value between 0 and 1 that gives the most significant results on the data, depending on the training dataset. The results for every value of *α* are shown in Supporting Information. A distribution of *η* for a particular connectome type that is shifted to the positive values means that the similarities between the connectomes have globally increased when applying the permutations identified by a given optimization strategy (HA, WBA, and WBANC) using a given training dataset and for a given value of *α*. Statistically significant increases in similarity are indicated with a star above the distributions in Figures 3 and 4, while significant decreases are marked with a negative star.

### Alignment Using Resting-State Connectomes

The permutation matrices are first identified on the primary training dataset consisting of SC and rs-FC_1_ (see Figure 2C). The error criterion *η* is computed on the training dataset, thus on SC, and rs-FC_1_. The criterion *η* is also computed on rs-FC_2_ and t-FC to assess the validity of the identified permutations. The distributions of the criterion *η* are plotted in Figure 3 for *α* = 0 (first column, alignment on rs-FC_1_ only), 0.3 (second column), and 1 (third column, alignment on SC only). For the specific cases: *α* = 0 and 1, the alignment criteria use only one type of connectome modality, for this training data, either rs-FC_1_ (*α* = 0) or SC (*α* = 1). The results for the 11 values of *α* are shown in the Supporting Information.

The *η* distributions for connectomes used in the optimizations (training dataset) are shown in dark colors (red for SC and dark green for rs-FC_1_), and in light colors for connectomes not used in the optimization criteria (light green for rs-FC_2_ and light blue for t-FC). For the single-modality alignment (*α* = 0 and 1), the permutations significantly align the connectome types on which they are optimized. In particular, for *α* = 0 the rs-FC_1_ are significantly aligned and for *α* = 1 the SC are significantly aligned, regardless of alignment strategies. Nonetheless, the alignment on SC only (*α* = 1) does not align rs-FC_1_ and conversely. Hence, the need for a multimodal alignment with an *α* value between 0 and 1 excluded. For *α* = 0.3, so for permutations identified on both SC and rs-FC_1_, the distributions of *η* for SC and rs-FC_1_ are shifted to the positive direction, meaning significant alignment of SC and rs-FC_1_ simultaneously for the three alignment strategies. In addition, for *α* = 0 and 0.3, the distribution of the rs-FC_2_ (light green) is also shifted to the positive direction for the three strategies, meaning a generalization of the identified permutations to rs-FC_2_. We also notice that when the results are significant, notably for rs-FC_1_ at *α* = 0 (dark green in the left column) and for SC at *α* = 1 (red in the right column), the WBA strategy (second row) shifts the distributions more toward positive directions than the HA strategy (first row). This means that the inter-hemispheric information helps the alignment process as expected. For the WBANC strategy, the permutations have a lower impact on the shifts of the distributions, but the results are still significant, meaning that even with the constraints to spatially adjacent regions, we can find permutations that decrease the distances between the connectomes, for SC and rs-FC_1_ simultaneously and that also improve the matching for rs-FC_2_.

### Alignment Using Task Connectomes

Secondly, permutation matrices are identified on the second training dataset, so on SC and task t-FC. The error criterion *η* is thus computed on this training dataset (SC and t-FC). The same criterion *η* is computed on rs-FC of both runs to assess the validity of the identified permutations. The distributions of the criterion *η* are plotted in Figure 4 for the three strategies and *α* = 0 (left column, alignment on SC only), 0.2 (middle column), and 1 (right column, alignment on t-FC only). The results for the 11 values of *α* are shown in the Supporting Information.

The *η* distributions for connectomes used in the optimizations are shown in red for SC and blue for t-FC, and in light green for connectomes not used for the optimizations, in this case, rs-FC_1_ and rs-FC_2_. For *α* = 0 and 1, the permutations significantly align the t-FC and SC, respectively, but do not generalize to the other connectome modalities of the training data (SC or t-FC). In other words, permutations identified on SC, when applied to t-FC do not decrease the distances between the connectomes, and the converse. However, for *α* = 0.2, using the identified permutations on both SC and the t-FC, the distributions of *η* for both SC and t-FC are shifted to the positive directions. We notice that for *α* = 0, so for an alignment considering only t-FC, the distributions of the rs-FC of the two runs (light greens) are also shifted to the positive directions, meaning a generalization of the alignment to resting-state connectomes for the three strategies. As for the results on the previous training dataset, when results are significant, the distances after permutations are always lower with the WBA strategies than with the HA strategy, meaning interhemispheric connectivities still inform the alignment. For *α* = 0.2, the three strategies allow alignment of simultaneously SC and t-FC. The more interesting result is that for any *α* values, only the WBANC strategy enables alignment of the four connectome modalities at the same time. Here, in particular, for *α* = 0.2 (third row and second column), the four distributions are shifted to the positive values.

### Constraints’ Effects on Distributions of Permutations

**Figure 5.**
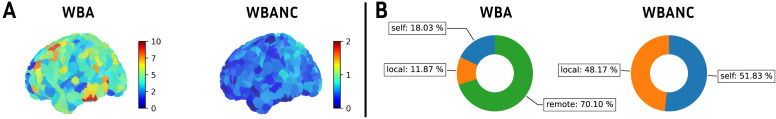
Distributions of the permutations for the optimization with constraints on intra-hemispheric permutations and neighbor’s permutations. A shows the averaged distance with the permuted regions for each region on brain maps for the alignment with constraints on intra-hemispheric regions (left) and constraints on neighbor regions (right). The distance is computed as the minimum path length between two regions on the graph of spatially adjacent regions. B shows the distributions of the permutations into three categories. The labels “self,” “local,” and “remote” stand for self-permutations (no permutations), local permutations (permutations between neighbors’ regions), and remote permutations (permutations between non-neighbor regions), respectively. The left pie chart corresponds to the optimizations with constraints on intra-hemispheric regions, and the right one on neighbors’ regions. The values shown are averaged values over all *α* values and for the training dataset made of SC and rs-FC_1_.

The regularization term directly influences the distance between the permuted regions in the WBA and WBANC strategies. We compare the identified permutations of the two approaches in terms of spatial distances between the permuted regions. The spatial distance between two permuted regions is calculated as the minimum path length between the two regions on the graph of spatially adjacent regions. In particular, self-permutations have a distance of 0, and permutations of spatially adjacent regions have a distance of 1. In Figure 5A, we can observe the averaged distance with the permuted region for every region on a brain map, for the WBA (left) and the WBANC (right). HA and WBA have similar spatial distances. For that reason, we only compare the values between WBA and WBANC. In the WBANC, permutations of remote regions are forbidden, while in the WBA, permutations of regions are only limited to within hemispheres. In Figure 5B, we show a pie chart of the distributions of the entire set of permutations. Permutations of regions are classified into three categories: self-permutation (region not permuted), local permutation (region permuted with a neighbor’s region), and remote permutation (region permuted with a region within the same hemisphere that is not a neighbor). The pie chart indicates that there are significant amounts of permutations performed, notably remote ones when no spatial constraints are used (Figure 5B, left). The use of the constraint to spatially adjacent regions has indeed restricted the permutations to local ones (Figure 5B, right), but there are still an important number of permutations with the constraints (48.17%).

## DISCUSSION

The results on the alignment of different types of brain connectomes highlight the complexity of aligning brain regions. Viewing the brain networks from different perspectives changes the alignment results, and aligning the brain regions with only one modality network does not allow for alignment on other brain network modalities (structural and functional). However, using a multimodal alignment optimization permits identifying permutations supported by both structural and functional connectivities, with functional connectivity being from either task or resting-state. Furthermore, the permutations found for the alignment on the rs-FC_1_, increase the similarities between the rs-FC_2_. This suggests that the permutations of regions are not purely noise alignment because they are reliable on the rs-FC_2_.

It seems that the generalization of the permutations identified on task connectomes to the resting-state ones is easier than the converse. Cole, Bassett, Power, Braver, and Petersen (2014) have shown that task functional networks are shaped primarily by an intrinsic network architecture that is also present in resting-state and secondarily by a limited set of task-general and task-specific evoked connectivity changes. The presence of the resting-state intrinsic network in task connectomes can explain the generalization to resting-state connectomes, while the permutations identified on resting-state miss the task-related networks and thus do not generalize easily to the task.

Overall, alignments do not generalize well across modalities (SC and FC). Alignment using the same modality (in our case different FC) generalizes better between them, and this is further improved when aligning between data acquired with the same protocol (in our case rs-FC).

Despite the quantitative noise removal of MRI data, the SC and FC probably still contain non-negligible noise, which could lead to overfitting of some permutations. For example, the permutations resulting from the alignment on rs-FC of run-1 (with alpha = 0) increase the similarity of the connectome when applied to the rs-FC of run-2, but the increase is less pronounced, meaning some subsets of permutations have possibly been overfitted. The lack of generalization between SC and FC is more complex. While spurious permutations resulting from the alignment on a single modality presumably exist, we believe that SC and FC reveal very different information about the brain networks. In the article of Deslauriers-Gauthier et al. (2020), they show that SC and FC have a correlation of only 0.25% on average, which argues for the need for a multimodal alignment.

To reinforce the relevance of the permutations, we tested the multimodal alignment using either rs-FC_1_ or rs-FC_2_. This allows us to observe the difference between the resulting permutations from the two sets of connectomes (with the conditions that give the best results of generalization, i.e., *α* = 0.3). We align the connectomes of 50 subjects with the mean connectomes using either rs-FC_1_ or rs-FC_2_. On average, 84% of the labels are exactly at the same location for the two alignments using either rs-FC_1_ or rs-FC_2_ (including permuted and nonpermuted labels). When restricting to permuted labels only, 52% of the labels were identically placed by both permutations. The different permutations across the two runs can be accounted for partly by the nonoptimality characteristic of the FAQ algorithm, partly by the noise of BOLD signals, and partly by the significant inter-session variability of rs-fMRI widely studied (S. M. Smith et al., 2005; Wens et al., 2014; Zandbelt et al., 2008).

The widths of the distributions of *η* in Figures 3 and 4 show the great variability in the alignment results, meaning some subject connectomes align better to certain subjects. The results of Rasero, Diez, Cortes, Marinazzo, and Stramaglia (2019) indeed suggest that there are population subgroups of functional networks as clustering algorithms find natural clusters in a functional connectome set. Interestingly, the WBANC strategy has a lower variability of *η* values than other strategies (more peaked distributions), suggesting that restricting the set of permutations leads to a more homogeneous group of connectomes. To study this phenomenon, we calculated the mean *η* value per subject of reference, instead of on all the pairs of subjects. The standard deviation of these means across subjects of reference is strictly lower for the WBANC than for the WBA (1.58 vs. 6.89 on SC for *α* = 0.3, using the first training dataset). This means that some subjects are better references, in the sense that they decrease more significantly the connectome distance when chosen as a reference, but it also suggests that the restriction to the spatially adjacent regions decreases this variability across the references. The selection of a reference is a common problem in neuroscience, encountered, for example, in registration. The typical choices are a subject of the cohort or a template such as the MNI152. In our case, generating a template would require constructing group-averaged SC and FC. However, we cannot simply average the group connectomes, as we do not assume a perfect matching of the labels across subjects. We could have computed the Frechet mean of all the connectomes, which consists of the iterative process of computing the Euclidean mean of the subject’s connectomes and aligning every connectome to this intermediate mean, until convergence (Calissano, Feragen, & Vantini, 2024). This would have given a graph-unlabeled mean, where each region does not correspond to a single label anymore, therefore losing the interpretability of the labels. Using a given subject as reference allows us to get a direct mapping between real subjects, where we preserve the specific meaning of each region in the graph.

Regarding the frequency of the permutations, the pie chart illustrated in Figure 5B shows the great number of permutations that are performed on a parcellation with such tiny regions (1,000 regions within the cortex). These results strengthen the need to consider the spatial variability across subjects when using fine-grained parcellation. The need for an alignment procedure depends on the granularity of the parcellation. Indeed, for broad regions, there is no need for alignment. In the article of Calissano, Papadopoulo, et al. (2024), they show that permutations start to arise for parcellation granularities of more than 400 regions over the cortex.

Our alignment criteria currently use two types of connectomes, and we have shown that this is not enough to align perfectly with every other connectome modality. Including many connectome modalities directly in the alignment process, such as SC, rs-FC, and t-FC, might be judicious to ensure the alignment of all brain networks, as we have shown it is mathematically possible with our improved version of the FAQ algorithm. This would imply having other hyperparameters to tune, and this would require external data not seen in the optimization to validate the identified permutations, which are not available in the HCP dataset.

We investigated how the permutations resulting from the alignment of SC and rs-FC_1_ affect the activation maps computed from t-fMRI for different motor stimuli. We observed that these permutations do not significantly increase or decrease the similarity across subjects of these activation maps. This result reflects the complexity of the relationship between a brain region’s network role and its task-specific activation, which is not straightforward. Under specific hypotheses, we can show that aligning the connectome computed from a task-fMRI is more similar to aligning the *correlation* matrices of the activation estimates of several stimuli, which does not imply an increase in the similarity of the parameter estimates’ maps directly. The demonstration is in the Supporting Information. One way to bridge this gap is to add another term to the objective function of the alignment that includes direct alignment of activation maps. Future work could include modifying the alignment framework to account for the increased similarity of different maps, such as activation maps or other properties, like cortical thickness maps.

The main argument for the use of the spatial constraint lies in the limitations of the functional view of the brain networks. Functional connectivity computed using Pearson’s correlation coefficient is a statistical approximation of the effective connectivity between gray matter regions and represents direct and indirect connections using intermediary regions (Sporns, 2011). As a consequence, more regions are considered connected than in structural networks, and remote regions with similar functional connectivity profiles might be exchanged. Performing brain graph alignment on the sole basis of functional connectivity could lead to spurious alignment with meaningless permutations. As the brain cortex organization is globally similar across subjects (Pessoa, 2014), we avoid absurd permutations of remote regions by using a regularization term in the optimization process of alignment, which acts as a constraint to restrict the set of permutations.

Using the WBANC method, we restrict permutations to neighboring regions that depend on the parcellation size. We could imagine using the spatial distance between regions to construct a regularization matrix, to favor permutations of spatially close regions rather than strongly restricting permutations as our optimization criterion does.

Our alignment framework has been used for individuals from the same species but could be applied across species, similarly to the work of Mars et al. (2016), with the limitations that we need to have the same number of regions across the cortex for all the species. One possibility would be to combine our network alignment with the clustering of regions to map several small regions to a different number of regions.

Analysis tools for groups of subjects generally do not account for spatial variability. To consider it, the usual tools should be revisited, as was the case in fMRI analyses, where some methods account for spatial variation between subjects to extract common fMRI patterns (Haxby et al., 2011; Michael, Anderson, Miller, Adalı, & Calhoun, 2014; Takerkart, Auzias, Thirion, & Ralaivola, 2014). Our alignment approach is a step toward correcting for inter-subject variability before using the classical tools of connectome analysis at the group level. For between-group comparisons (e.g., control vs. patient groups), the selection of the reference is a critical concern, as we would not want to decrease the between-group differences we are looking for. Thus, it remains to be determined whether a single reference should be used across all groups or whether separate references should be selected for each group.

Future work should include finding a way to identify the subjects who are the best references for the alignments, as we can only identify them post hoc. We also plan to use our alignment framework as a preprocessing step before group studies, to observe to what extent inter-subject variability impacts the results of the state-of-the-art group analysis tools. As an example, we will align the brain regions before attempting to predict FC from SC. Indeed, it remains challenging to produce a good estimator of functional networks from structural ones (Deslauriers-Gauthier et al., 2020; Smolders et al., 2024), possibly because current methods do not capture inter-subject variability when constructing structure–function mapping of subject groups.

## CONCLUSION

In this work, we propose a multimodal alignment algorithm that simultaneously increases the similarity of SCs and FCs across subjects. In comparison to previous work, our approach enforces anatomical constraints when permuting regions, leading to more robust permutations across connectome modalities. Beyond the novelty of the alignment framework, the results reveal substantial variability in connectivity at a fine-grained scale. Therefore, we recommend that researchers take spatial inter-subject variability into account when studying fine-grained connectivity.

## ACKNOWLEDGMENTS

The authors are grateful to the OPAL infrastructure from Université Côte d’Azur for providing resources and support.

Data were provided by the Human Connectome Project, WU-Minn Consortium (Principal Investigators: David Van Essen and Kamil Ugurbil; 1U54MH091657) funded by the 16 National Institutes of Health (NIH) Institutes and Centers that support the NIH Blueprint for Neuroscience Research; and by the McDonnell Center for Systems Neuroscience at Washington University.

## SUPPORTING INFORMATION

Supporting information for this article is available at https://doi.org/10.1162/NETN.a.514.

## AUTHOR CONTRIBUTIONS

Yanis Aeschlimann: Conceptualization; Investigation; Methodology; Software; Visualization; Writing – original draft; Writing – review & editing. Anna Calissano: Conceptualization; Writing – review & editing. Théodore Papadopoulo: Methodology; Supervision; Writing – review & editing. Samuel Deslauriers-Gauthier: Conceptualization; Methodology; Supervision; Writing – review & editing.

## FUNDING INFORMATION

Yanis Aeschlimann, Université Côte d’Azur (https://dx.doi.org/10.13039/501100013371).

## Supplementary Material



## TECHNICAL TERMS

Parcellation: Distinct partitions in the brain.
Graph similarity: In our study, similarity is defined by the Frobenius distance between the two graphs represented as square matrices.
Graph alignment: The problem of finding a bijective mapping across the vertices of two graphs with the same number of nodes.
Permutation matrix: Is a square binary matrix that has exactly one entry equal to 1 in each row and each column, with all other entries equal to 0.
Assignment problem: In a weighted bipartite graph, find a matching in which the sum of weights of the edges is minimum.
NP-hard: Informally, an NP-hard problem cannot be solved by testing every possibility, because it would require an unrealistic amount of computing capacity.
Conditional gradient descent: An Iterative first-order optimization algorithm for constrained convex optimization.
Bistochastic matrix: A square matrix of nonnegative real numbers, for which the sum of elements across every row and column is 1.
Tractogram: A set of streamlines, representing white matter bundles of the brain.
