# Supplementary material for: Inter-individual alignment of multimodal brain networks with anatomical constraints
Source: Netw Neurosci. 2026 Jan 28;10(1):137–57. doi: 10.1162/NETN.a.514 (PMC12956296; doi:10.1162/NETN.a.514)
Supplement: Supplementary file 1 [file netn-10-1-137-s001.pdf]

Journal: **NETWORK NEUROSCIENCE**

RESEARCH

SUPPLEMENTARY MATERIAL: INTER-INDIVIDUAL ALIGNMENT OF MULTIMODAL BRAIN NETWORKS WITH ANATOMICAL CONSTRAINTS

Yanis Aeschlimann[1], Anna Calissano[2], Theodore Papadopoulo[1],

and Samuel Deslauriers-Gauthier[1]

[1]Université Côte d'Azur, Inria, Sophia-Antipolis, France

[2]Department of Mathematics, Imperial College London, London, United Kingdom

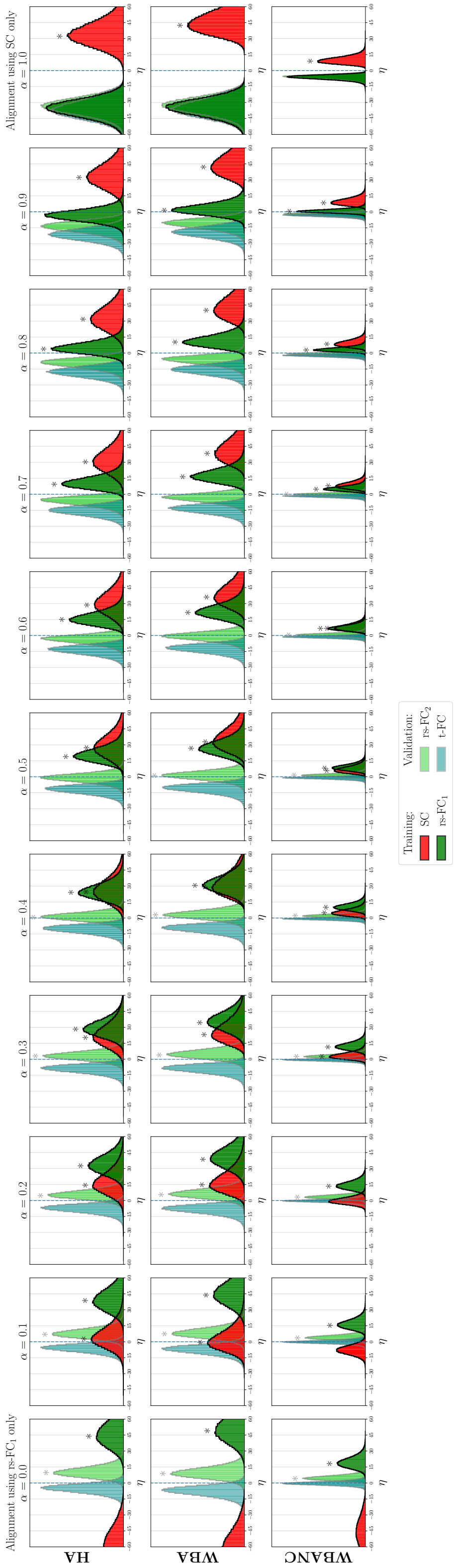

Figure 1: Distributions of the difference between the connectomes' distances respectively before and after the alignment on structural and resting-state functional connectomes of the first run, for the three strategies: HA (top row), WBA (middle row), and WBANC (bottom row) and every value of α between 0 and 1 with a step size of 0.1.

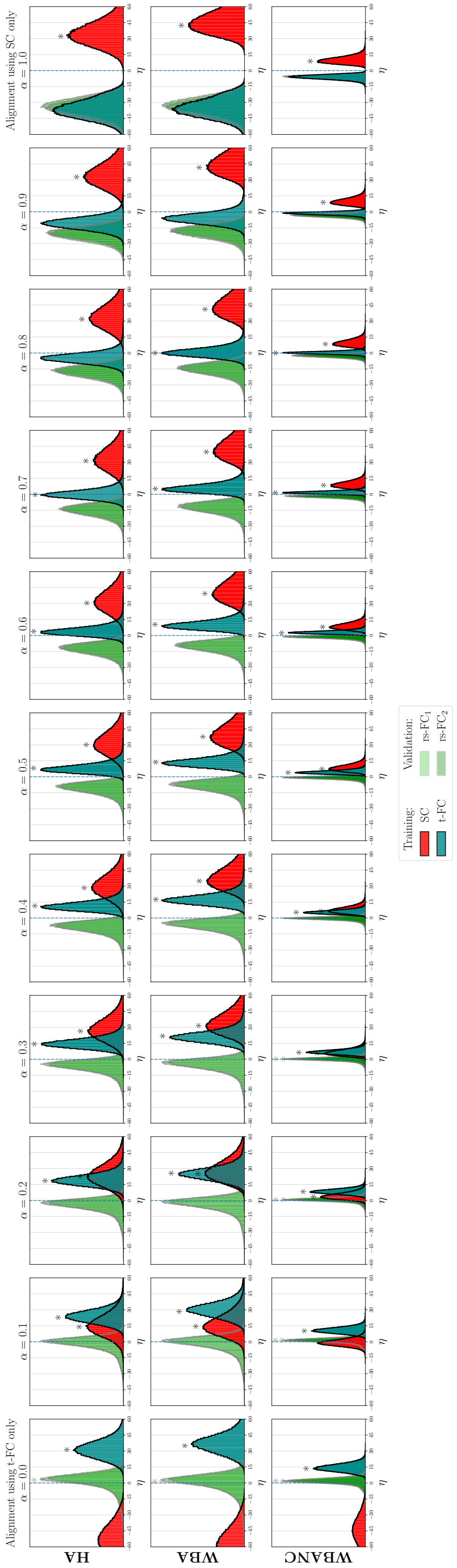

Figure 2: Distributions of the difference between the connectomes' distances respectively before and after the alignment on structural and task functional connectomes, for the three strategies: HA (top row), WBA (middle row), and WBANC (bottom row) and every value of α between 0 and 1 with a step size of 0.1.

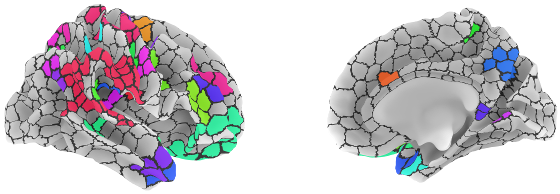

(a)

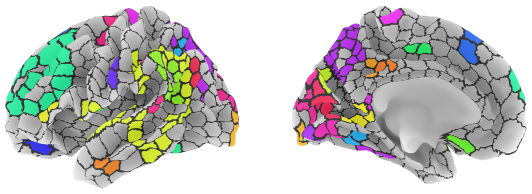

(b)

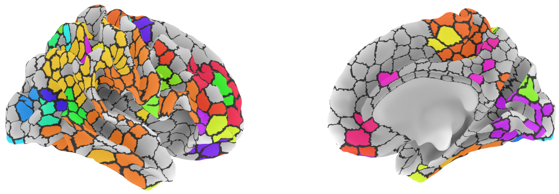

(c)

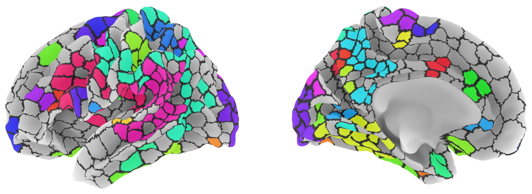

(d)

Figure 3: Four different examples of alignment of subjects using the WBANC strategy. The permutations of regions occur in cycles of spatially adjacent areas. Each color corresponds to a cycle of permutations.

Alignment of connectome versus alignment of activation maps

A functional connectome is computed as

$$C = AA^T \tag{1}$$

where A is an $N_{\text{regions}} \times t_{\text{samples}}$ matrix containing the averaged normalized BOLD signals for each brain region. Assuming a very simple general linear model (GLM), we can compute the activation maps. We have

$$A^T = G\hat{\beta}, \tag{2}$$

neglecting the noise and the BOLD normalization. G is the design matrix that contains the regressors (i.e., stimuli convolved with the hemodynamic response function and noise regressors), and $\hat{\beta}$ are the estimated strengths of the regressors for all regions.

Aligning the functional connectomes minimizes

$$\|PAA^TP^T - A_rA_r^T\| = \|P\hat{\beta}^TG^TG\hat{\beta}P^T\hat{\beta}_r^TG^TG\hat{\beta}_r\|. \tag{3}$$

With the strong assumption that G is semi-orthogonal (which is not in practice, at least due to the overlap of the regressors), i.e. $G^TG = I$, this simplifies to minimizing $\|P\hat{\beta}^T\hat{\beta}P^T - \hat{\beta}_r^T\hat{\beta}_r\|$, which is the alignment of the *correlation* matrices of the estimators $\hat{\beta}$.
